# RXRα provokes tumor suppression through p53/p21/p16 and PI3K-AKT signaling pathways during stem cell differentiation and in cancer cells

**DOI:** 10.1038/s41419-018-0610-1

**Published:** 2018-05-10

**Authors:** Rui Zhang, Hui Li, Shuangshuang Zhang, Yujie Zhang, Nan Wang, Hao Zhou, Hongpeng He, Guang Hu, Tong-Cun Zhang, Wenjian Ma

**Affiliations:** 1 College of Biotechnology, Tianjin University of Science and Technology, 300457 Tianjin, China; 2 Institute of Biology and Medicine, Wuhan University of Science and Technology, 430081 Wuhan, China; 3 Qilu Institute of Technology, 250200 Shandong, China

## Abstract

The retinoid X receptor alpha (RXRα) is an important therapeutic target impacting diverse biological processes. Activation of RXRα is known to suppress cancer cell growth. However, the cellular mechanism has been elusive. In the present study, we addressed its role during stem cell differentiation and the underlying connections with carcinogenesis. RXRα was significantly upregulated following the differentiation of human mesenchymal stem cell (hMSC) toward the formation of endothelial cell (EC). However, overexpression of RXRα in hMSC provoked a senescence-like phenotype accompanied by the elevation of tumor suppressor p53, p21, and p16. Consistently, RXRα level was suppressed in cancer cells (~five times lower compared to differentiated hMSC), and its elevation could inhibit the proliferation, migration, and angiogenesis of cancer cells. We further demonstrated that these inhibitory effects were related to RXRα’s interaction with estrogen receptor α (ERα) as well as EGF and ANGPTL3 through modulating PI3K/AKT signaling pathway by AKT and FAK phosphorylation. Moreover, RXRα inhibited glycolytic metabolism in cancer cells, which might be underlying its inhibition of differentiation and carcinogenic features. These data suggest that RXRα acts as a suppressor rather than a driving force during stem cell differentiation, and unbalanced RXRα can trigger multiple yet connected signaling pathways in preventing carcinogenesis.

## Introduction

Cancer cells and stem cells share similarities, such as the ability of self-renewal and the potential for differentiation^1^. It has been proposed that cancer cells might be originated from certain stem cells with malignant mutations termed cancer stem cells (CSCs)^2, 3^. CSCs showed higher resistance to various commonly used chemotherapeutic treatments^4–7^, and are believed to be a driving force for tumor recurrence and metastasis^8–10^.

The multistep process of cancer progression requires genome alterations that accumulated with cell proliferations and divisions^1^. The occurrence rate is low in normal cells owing to the limited number of cell divisions. However, the probability of accumulating multiple mutations in stem cells could be greatly elevated with their unlimited dividing capacity^9^. Tomasetti et al. reported recently that the occurrence of cancer is strongly correlated with the number of stem cell divisions in different tissues, which extended over five orders of magnitude based on the analysis of 31 cancer types^11^. This provided a strong support to the cancer stem cell hypothesis and emphasized the importance of cell division during carcinogenesis.

Considering that differentiated cells rarely proliferate, modulation of the cellular mechanisms to prevent stem cells from differentiation but retain at certain stages with proliferation capacity might be required in order to obtain sufficient genetic alterations for carcinogenesis. The cross talk between stem cell differentiation and carcinogenesis has been largely unknown. It is interesting to find out whether modulating stem cell differentiation could facilitate the conversion of normal stem cells into CSCs.

In the present study, we have addressed the role of retinoic acid receptor α (RXRα) in attempting to identify the cellular components that may impact both stem cell differentiation and neoplastic transformation. RXR is a family of nuclear receptors implicated in the control of a variety of physiological processes such as lipid and glucose metabolism and immune responses^12, 13^. Some RXR isoforms have even been shown that can facilitate the induction of pluripotent stem cells^14, 15^. Being the most abundant and functional isoform of RXR in various cell types, RXRα is a central transcriptional regulator in modulating gene expression by hetero-dimerization with other nuclear receptors^16^.

Regulation of RXR by natural and synthetic ligands (e.g., vitamin A and retinoic acid derivatives) is known to inhibit cell proliferation and has been used to treat cancers^17–19^. However, the underlying mechanism is not fully understood. Here, using human mesenchymal stem cells (hMSC) as a model for stem cell differentiation, and by comparing with cancer cell lines, we sought to determine the cellular consequences of modulating RXRα during cell differentiation as well as the possible connections with carcinogenesis.

## Results

### RXRα was increasingly expressed during the differentiation of hMSC into epithelial cells but was generally suppressed in cancer cells

**Fig. 1:**
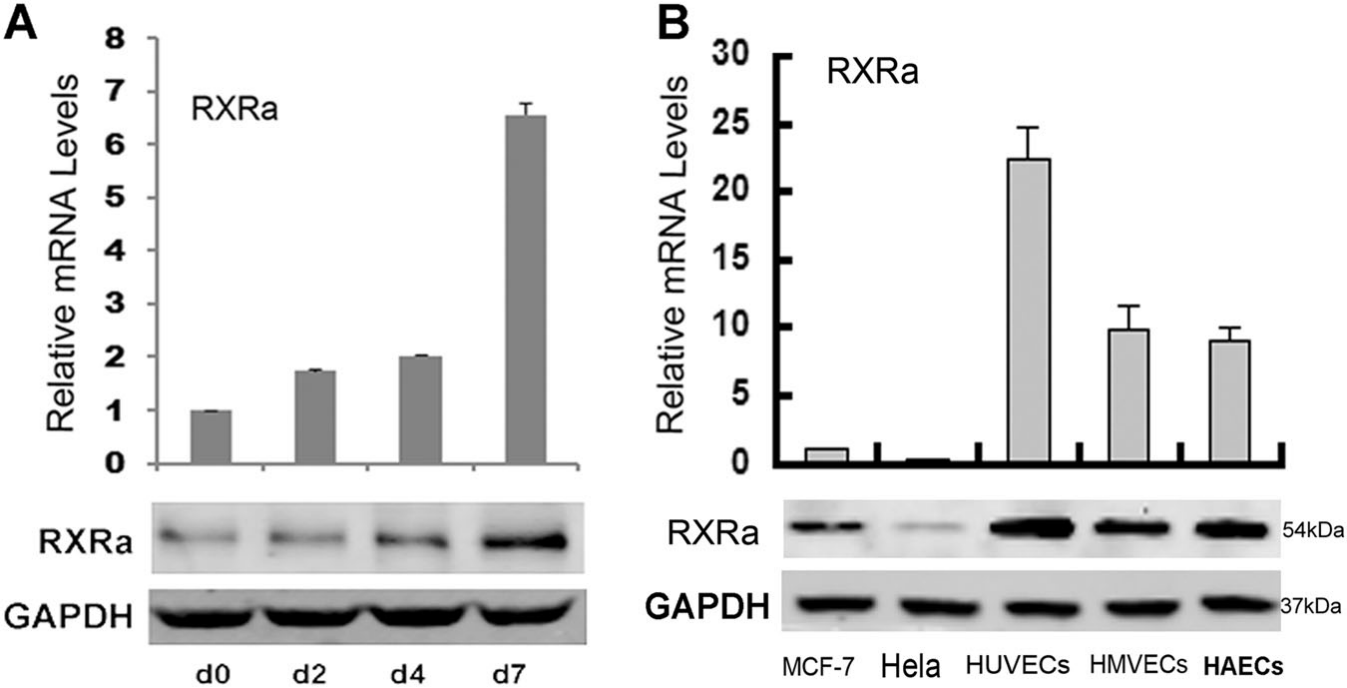
RXRα expression during differentiation of hMSCs in normal epithelial cells and cancer cells. **a** The expression of RXRα in hMSCs cultured in EDM at days 0, 2, 4, and 7. **b** The expression of RXRα in cancer cells MCF-7 and HeLa, endothelial cells HUVECs, HMVECs, and HAVECs. The expression was determined by qRT-PCR and western blotting (*P* < 0.01, *n* = 3)

Tumor progression requires the activation of an “angiogenic switch” to drive the formation of new vessels, which involves the formation of new endothelial cells^20^. Endothelial cells can be differentiated from hMSCs, and it has been used for adult vascular repair and regeneration therapies^21^. To investigate what role RXRα plays during this process, we first determined the expression of RXRα during the differentiation of hMSCs toward endothelial cells. As shown in Figure 1a, RXRα protein level was increased in a time-dependent manner during differentiation, showing a sharp increase (~seven fold) at day 7 when endothelial cells were formed. In contrast, the RXRα levels determined in various human cancer cell lines were much lower. Of eight cancer cell lines that were tested (HeLa and MCF-7 were shown in Figure 1 as representatives), RXRα levels were found to be 5–20 times lower than that in various endothelial cell lines (HUVECs, HMVECs, and HAVECs) that hMSC can differentiate into as well as in the non-transformed breast cell line MCF10a (used as control for MCF-7) (Fig. 1b and Supplemental Fig. S1). These data on the one hand confirmed that RXRα played an important role during cell differentiation. On the other hand, it raises an interesting question—does suppressing RXRα during stem cell differentiation facilitates the process of carcinogenesis, which may account for its low expression level in cancer cells?

### Overexpression of RXRα in hMSCs inhibited proliferation, activated tumor suppressor genes, and induced senescence-like cell phenotype

**Fig. 2:**
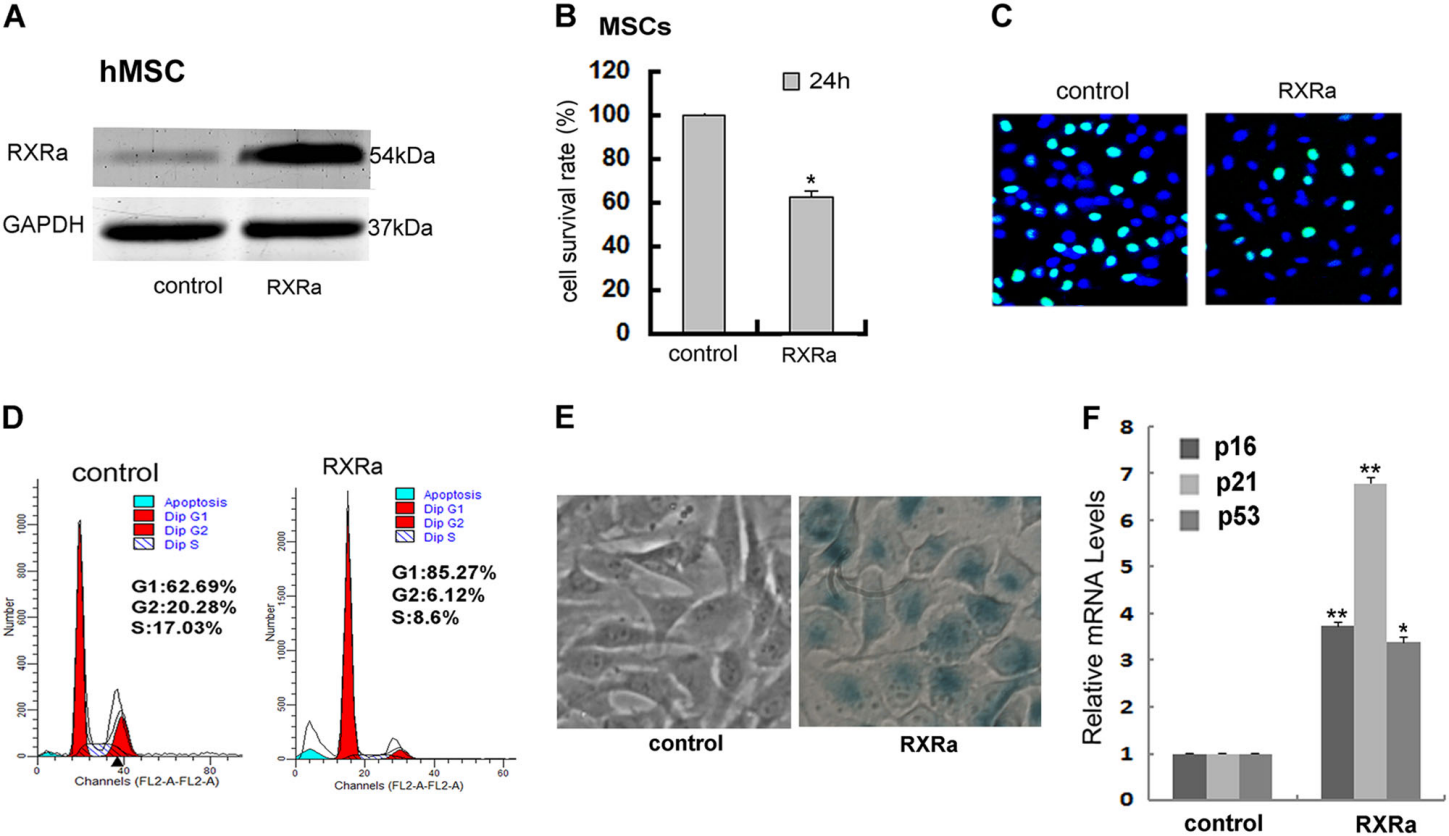
Overexpressing RXRα in hMSCs inhibited proliferation, activated tumor suppressor genes, and induced senescence-like cell phenotype. **a** Forty-eight hours after transfection with RXRα plasmid, RXRα protein level was determined by western blot. **b**, **c** Cell proliferation was assessed by MTT (*P < 0.05) and EdU assay (quantification was performed by counting the positively stained cells in three randomly selected areas). **d** Flow cytometry detection of the cell cycle distribution. **e** β-galactosidase staining assay of hMSCs. **f** The mRNA levels of p16, p21, and p53 determined by qRT-PCR (*P < 0.05; **P < 0.01, *n* = 3). All measurements are shown as the means ± SD from three independent experiments

Since RXRα was expressed at a relatively low level in hMSCs compared to differentiated cells, we first asked whether ectopic overexpression of RXRα in hMSCs would speed up the differentiation process. Interestingly, overexpressing RXRα in hMSCs (nine times increase compared to the basal level of non-transfected control cells, Fig. 2a) did not elevate the differentiation process. Instead, it induced phenotypic changes reminiscent of cellular senescence. As determined by MTT and EdU analysis, hMSCs transfected with RXRα exhibited a decrease in cell growth rate (Fig. 2b–c). The proportion of cells in G0/G1 phases was significantly increased and the proportion of cells in S phase was decreased. These data suggest that RXRα overexpression in hMSCs inhibited proliferation by inducing cell cycle arrest in the G0/G1 phase (Fig. 2d). In addition, positive staining of the senescence-associated β-galactosidase, a widely used marker for cellular senescence^22, 23^, was observed in hMSCs after transfection with RXRα (Fig. 2e). Furthermore, the expression of major tumor suppressor genes p53, p21, and p16 were all induced in hMSCs when RXRα was overexpressed, especially the level of p21, which was increased by seven times (Fig. 2f). Overall, these results indicate that the elevated level of RXRα observed during the differentiation of hMSCs is not a driving force facilitating the progression of differentiation. In fact, it negatively controls cell proliferation, and overexpression might be even harmful to cells at an early stage of cell differentiation.

### RXRα overexpression in cancer cells inhibited cell proliferation, invasion, and angiogenesis

To understand why RXRα was suppressed in cancer cells, we overexpressed it in MCF-7 cells. Consistent with the activation of tumor-suppressing mechanism described above, overexpression of RXRα showed some inhibitory effects on cell proliferation, migration, and tube formation capacity as described below.

**Fig. 3:**
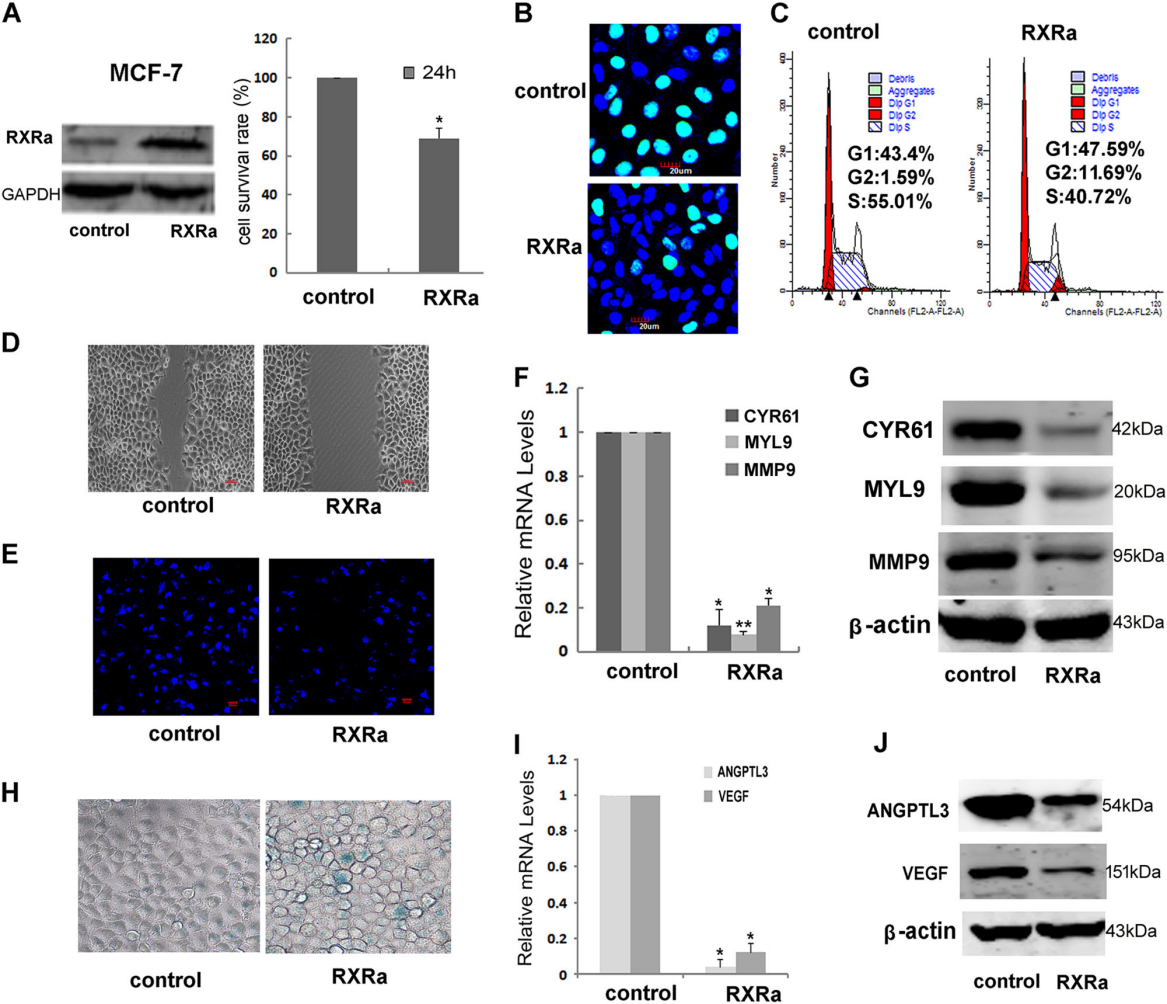
RXR overexpression in cancer cells inhibited proliferation, migration, and angiogenesis. **a** MTT assay and **b** EdU assay of MCF-7 transfected with RXRα after 48 h. **c** Flow cytometry detection of the cell cycle distribution of MCF-7 48 h after transfecting with RXRα. The migratory ability of MCF-7 overexpressing RXRα was determined via wound healing (**d**) and transwell chamber assays (**e**) as described in “Materials and methods”). The expression of Cyr61, Myl9, and MMP9 examined by qRT-PCR (**f**) and western blotting (**g**). **h** SA-β-galactosidase staining assay of parental and RXRα overexpression MCF-7 cells. **i** The expressions of ANGPTL3 and VEGF were examined by qRT-PCR (**j**) and western blotting (**k**) in MCF-7 overexpressing RXRα. Shown are the means ± SD from three independent experiments (*P < 0.05; **P < 0.01, *n* = 3)

The MTT and EdU data showed that RXRα overexpression exhibited a decrease in cell growth rate in MCF-7 (Fig. 3a–b). The proportion of cells at G0/G1 phases was evidently increased while the proportion of S phase cells was decreased, suggesting that RXRα overexpression also inhibited proliferation by inducing cell cycle arrest in G0/G1 phase (Fig. 3c). Unlike the transient overexpression of RXRα investigated in the present study, stable overexpression of RXRα in MCF-7 was previously reported to have little change on cell proliferation rates^24^. This discrepancy might be due to some unknown genetic adaptation changes, which need further clarification.

RXRα overexpression also showed strong impacts on cell migration as determined by wound healing^25^ and transwell chamber assays^26^. After creating a “scratch” in a monolayer of MCF-7 cells, the closure of the gap was determined after 24 h. As shown in Fig. 3d, the gap was significantly decreased when cells overexpressing RXRα. This was further confirmed by transwell assay. Cells seeded on the upper chamber of a transwell insert crossed the monolayer and migrated to the lower side of the membrane after 24 h of incubation, which were photographed and counted with confocal microscope. Evident migration and invasion were observed in control MCF-7 cells despite the known fact that this cancer cell line has low invasive potential. When overexpressing RXRα in MCF-7, however, the total number of cells that migrated across the membrane was reduced by more than 40% (Fig. 3e). Consistently, RXRα overexpression caused a significant reduction of the migration protein markers Cyr61, Myl9, and MMP9 in MCF-7 as determined by qRT-PCR and western blot (Fig. 3f–g).

Since the above genes are also known to be altered in senescence. It is interesting to know if senescence was induced in MCF-7 that overexpressing RXRα. Therefore, we determined senescence-associated β-galactosidase (Fig. 3h). Interestingly, the results were different from what we observed in hMSC and there were just some minor increase comparing to mock-treated control MCF-7 cell, suggesting that the activation of senescence responses by RXRα is altered in caner cells. Is it due to the ability of cancer cells in sequestrating RXRα to the splicing factor compartments (SFCs)^27^, and therefore leading to the loss of RXRα activity? This is an open question for future studies.

VEGF and the angiopoietin-like family member ANGPTL3 are important players during angiogenesis that are often upregulated by oncogene activation^28^. Consistently, our results showed that the expression of ANGPTL3 and VEGF were both significantly decreased when overexpressing RXRα in MCF-7 (Fig. 3i, j). It was reported that VEGF ligands can be sequestered by the matrix-degrading protease MMP9 leading to a positive feedback regulation^29^. The downregulation of MMP9 expression observed in current study is also in line with that notion. Collectively, the above results revealed that RXRα inhibits angiogenesis, either by itself or through interaction with other cellular components.

### RXRα downregulated angiogenesis-related markers partially through interacting with ERα

**Fig. 4:**
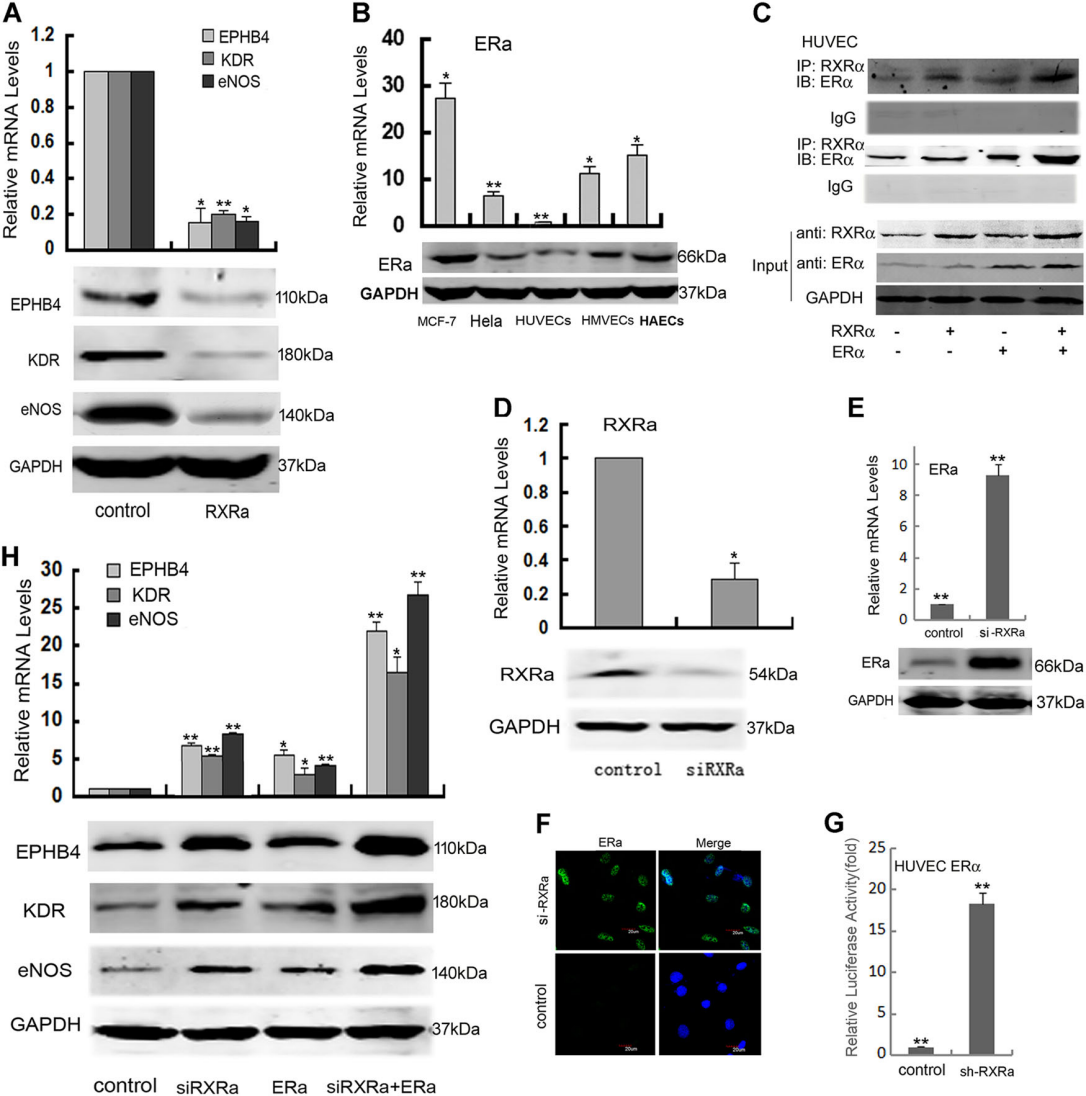
RXRα downregulated angiogenesis-related makers partially through interacting with ERα. **a** The expression of EPHB4, KDR, and eNOs in HUVECs overexpressing RXRα. **b** The expression of ERα in cancer cells MCF-7 and HeLa, endothelial cells HUVECs, HMVECs, and HAVECs. **c** Co-IP analysis of protein–protein interaction between RXRα and ERα (experiment was performed as described in “Materials and methods”). **d** Knockdown of endogenous RXRα in HUVECs with siRXRα. **e** The expression of ERα examined by qRT-PCR and western blotting in HUVECs following siRXRα knockdown. **f** Immunocytochemistry analysis of ERα in HUVECs. **g** Binding of RXRα on the promoter of ERα determined by luciferase assay. A plasmid containing the ERα promoter was transfected into COS-7 cells together with plasmids expressing ERa or/and RXRα, and luciferase activities were measured 24 h later. **h** The expression of EPHB4, KDR, and eNOs in HUVECs with siRXRα knockdown or/and ERα overexpression. For the measurements of luciferase reporter and mRNA level, the means ± SD from three independent experiments are shown (*P < 0.05; **P < 0.01, *n* = 3)

RXRα level appears to be tightly regulated in the cell. Although the level of RXRα was elevated together with increasing expression of differentiation markers (as is the case during hMSC differentiation), further increase in the level of RXRα led to suppression of the differentiation markers. This was observed when RXRα was overexpressed in HUVECs. As shown in Figure 4a, three angiogenesis-related marker genes (KDR, eNOs, and EPHB4) were all significantly decreased in HUVECs with RXRα overexpression as compared to that in control HUVECs.

RXRs normally exert their impact through interaction with other nuclear receptors. Noticing the promoter sequence that RXRα recognizes (AGGTCANNNAGGTCA) shares some similarity with that of estrogen receptor α (ERα) (GGTCANNNTGACCT), we wondered whether RXRα‘s participation in regulating angiogenesis has anything to do with ERα. Therefore, we investigated the effect of ERα on those differentiation markers in MCF-7 and HeLa, as well as endothelial cells HUVECs, HMVECs, and HAVECs. As shown in Figure 4b, the level of ERα was higher in MCF-7 than that in HUVECs, which is negatively correlated with RXRα (Fig. 1b). To investigate if RXRα and ERα have protein–protein interaction, a co-immunoprecipitation assay was performed. RXRα and ERα expression plasmids were co-transfected in HUVECs. Then, RXRα was immunoprecipitated using anti-RXRα antibody and an immunoblot was carried out with anti-ERα antibody (Fig. 4c, upper panel) and vice versa (Fig. 4c, middle panel). The results showed that RXRα and ERα were co-precipitated from the HUVEC cell extracts in either case, indicating that they do associate with each other. We also found that knockdown of endogenous RXRα by siRNA promoted the expression of ERα as determined by real-time PCR, western blot, and immunocytochemistry (Fig. 4e, f). To further confirm the effect of RXRα on ERa expression, luciferase assay was performed. A reporter construct containing the promoter of ERα was co-transfected with ERα and/or RXRα expression plasmids in COS-7 cells, followed by luciferase measurement. The results showed that RXRα could inhibit the activity of the ERα promoter (Fig. 4g).

To investigate the impacts of RXRα/ERα interaction on cell differentiation, the expression of angiogenesis markers KDR, eNOS, and EphB4 was determined in HUVECs with RXRα overexpression/knockdown and/or ERα overexpression (Fig. 4). RXRα overexpression strongly inhibited and its knockdown promoted the expression of differentiation markers (Fig. 4a, h). When combined with overexpression of ERα, a synergistic effect was observed, suggesting that the impact of RXRα on HUVECs differentiation is (at least partially) through interacting with ERα.

### Knockdown RXRα rebuilt adult cell’s capacity for migration and blood vessel formation, which was enhanced by ERα overexpression

**Fig. 5:**
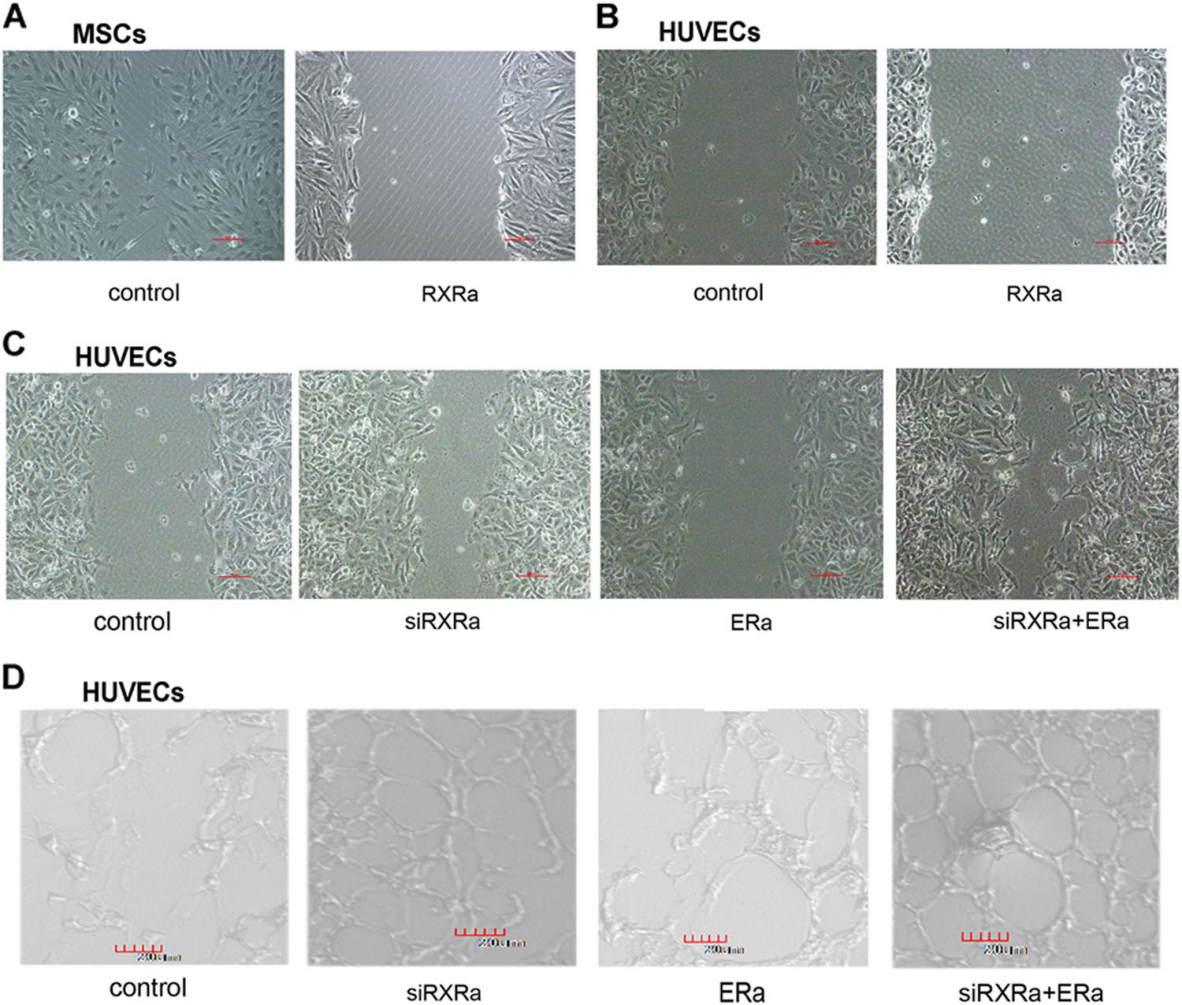
Knockdown of RXR rebuilt adult cell’s capacity for migration and blood vessel formation, which was enhanced by ER overexpression. **a**, **b** Determination of cell migration by wound healing in hMSCs and HUVECs overexpressing RXRα. **c** The migration of HUVECs with siRXRα knockdown or/and ERα overexpression. **d** Blood vessel formation determined by Matrigel assay in HUVECs with siRXRα knockdown or/and ERα overexpression (24 h culture in Matrigel)

Cancer cells typically develop alterations that facilitate invasion and metastasis^1^. To check the role of RXRα along this line, we determined its impact on cell migration and blood vessel formation. Migration was assessed by the wound healing assay. As shown in Figure 5a, overexpression of RXRα in hMSCs significantly inhibited cell migration. RXRα overexpression also mildly suppressed the migration capacity of HUVECs and its knockdown promoted migration (Fig. 5b, c). When knockdown of RXRα was combined with ERα overexpression, a significantly elevated cell migration was observed (Fig. 5c). To assess angiogenesis, we performed a Matrigel assay to determine the formation of tube-like structures on an extracellular matrix^30^. Compared to the control HUVECs that formed limited amount of capillary-like structures on Matrigel, RXRα knockdown and/or ERα increased the formation of these structures as evaluated by the total tube area and branching points (Fig. 5d). These results suggest that RXRα, through interacting with ERα, is also involved in controlling the process of cell migration and angiogenesis contributing to cancer cell invasion and metastasis.

### RXRα overexpression caused increased oxygen consumption and metabolism changes

**Fig. 6:**
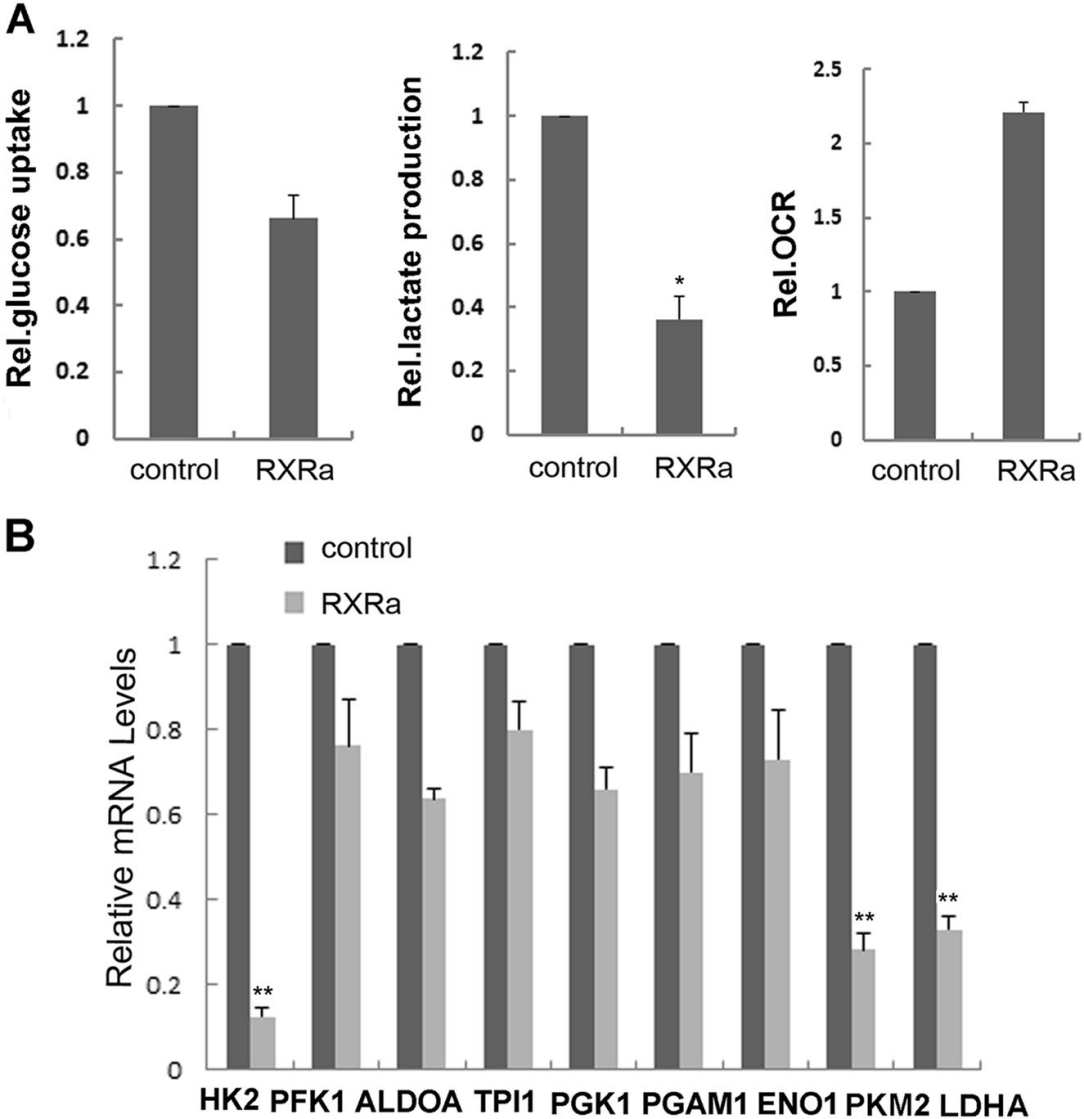
RXRα overexpression led to increased oxygen consumption and metabolic changes. **a** Cellular glucose uptake, lactate production, and oxygen consumption rates were measured in MCF-7 overexpressing RXRα using specified assay kit. **b** qRT-PCR analysis of HK2, PFK1, ALDOA, TPI1, PGK1, PGAM1, ENO1, PKM2, and LDHA in MCF-7 cells overexpressing RXRα

The growth of cancer cells involves not only a deregulated control of cell proliferation, but also the adjustment of metabolism to provide energy for growth and division^31^. To check the impacts of RXRα on metabolism reprogramming that might be related to its inhibitory effects on cell differentiation and migration, we investigated the metabolism changes in MCF-7 cell after overexpressing RXRα. Glucose, lactate, oxygen consumption, and the expression of metabolic enzymes such as HK2, PFK1, ALDOA, TPI1, PGK1, PGAM1, ENO1, PKM2, and LDHA were measured. As shown in Figure 6a, metabolic change did occur in RXRα overexpressing cells, which showed a lower rate of cellular glucose utilization and lactate production but increased rate of oxygen consumption. Consistently, we found that the mRNA levels of the key enzymes involved in glycolysis, including HK2, PKM2, and LDHA, were all decreased in MCF-7 treated with RXRα (Fig. 6b). These results indicated that the RXRα level affects glycolytic processes, as an antagonist for the “aerobic glycolysis” of cancer cells.

### Phosphorylation of AKT and FAK contributed to RXRα’s impacts on cell differentiation, migration, and angiogenesis

**Fig. 7:**
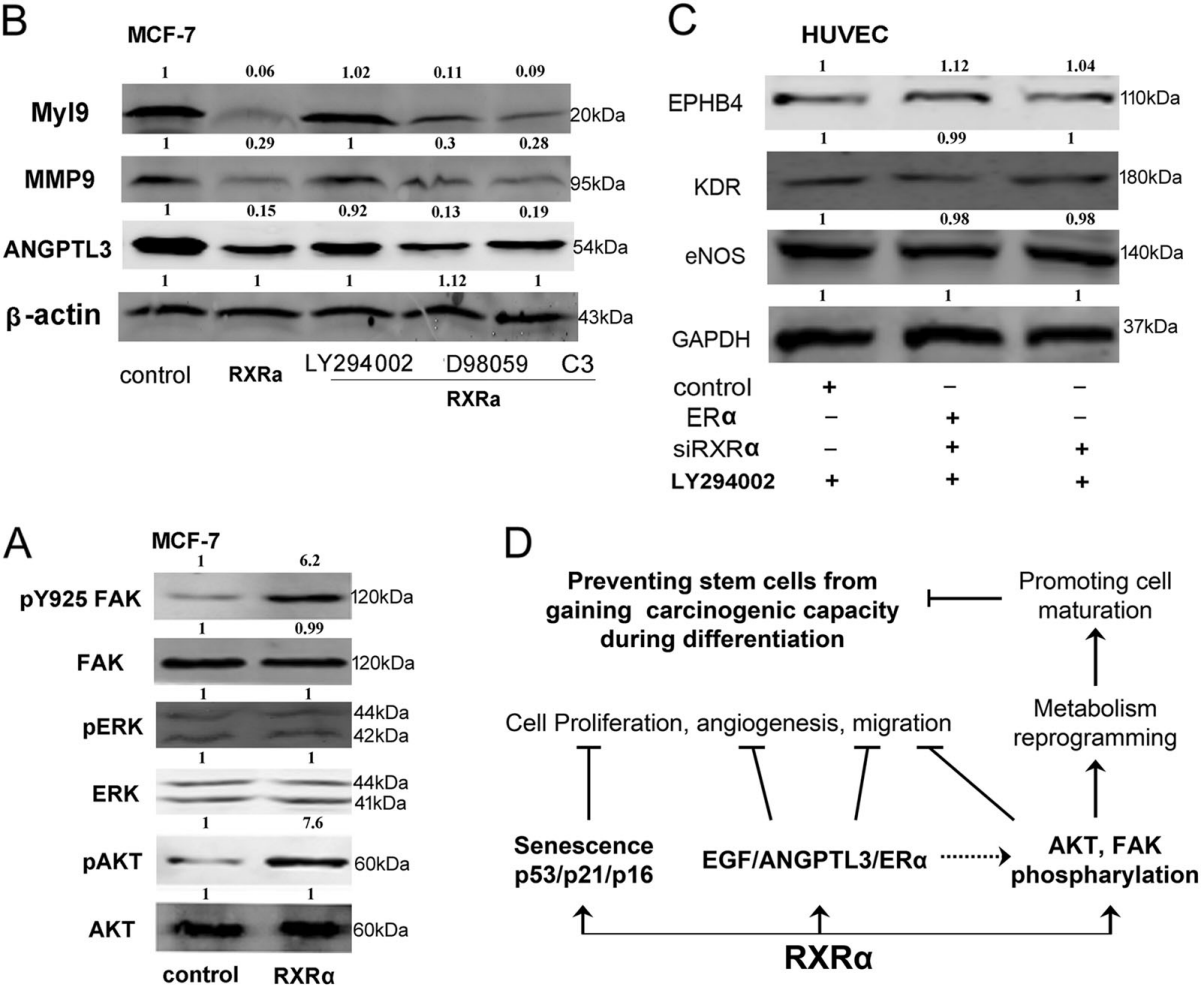
The signaling pathways underlying RXRα’s impacts on cell differentiation and carcinogenesis. **a** Western blot analysis of FAK, AKT, ERK, and their phospharylation in MCF-7 with RXRα overexpression. **b** Western blot analysis of migration markers Myl9, MMP9, and ANGPTL3 in MCF-7 cells pretreated with AKT inhibitor LY294002, ERK inhibitor PD98059, and the Rho-associated protein kinase (ROCK) inhibitor Y27632 (C3). **c** Western blot analysis of the expression of differentiation markers EPHB4, KDR, and eNOs in HUVECs with siRXRα knockdown or/and ERα overexpression after pre-treating with AKT inhibitor LY294002. **d** Scheme of signaling pathways that RXRα participates. RXRα inhibits cell proliferation, angiogenesis, and migration through interacting with VEGF, ANGPTL3, and ERα. It could trigger senescence-like tumor-suppressing mechanism by upregulating p53, p21, and p16 in stem cells. It also regulates/participates in metabolism reprogramming through phosphorylation of AKT and FAK. The multiple functions suggest that RXRα may be a key cellular component in protecting stem cells from carcinogenesis

To understand the downstream signal pathways underlying the diverse inhibitory effects of RXRα on differentiation, angiogenesis, and glycolysis, we determined whether it is related to PI3K/AKT or MAPK pathways, which are well known to transmit multiple cellular signals to influence the above processes ^32–34^. As shown in Figure 7a, MCF-7 cells with RXRα overexpression did not change the base level of AKT, but the phosphorylated form of AKT was evidently upregulated. The key component of MAPK pathway—ERK, however, did not show a change on either its base level or the phosphorylated form. These results indicated that RXRα exerted its cellular function through modulation of the PI3K/AKT pathway. We also determined the protein level of the focal adhesion kinase (FAK), which is a cytoplasmic non-receptor protein–tyrosine kinase that is tightly linked to the embryonic development and tumorigenesis^35^. FAK is known to integrate signals, such as growth factors and mechanical stress, to activate the P13K/Akt and Ras/MAPK pathway. As shown in Figure 7a, overexpression of RXRα led to the elevation of FAK phosphorylation at Tyr-925 (FAK-Y925). This phosphorylated form was reported to reduce cell migration and cell protrusion^36^.

To further confirm that RXRα’s suppression on cell migration was through the AKT pathway, we checked the migration protein markers Cyr61, Myl9, and MMP9 following RXRα overexpression in the presence of AKT inhibitor LY294002, ERK inhibitor PD98059 and the ras homolog gene family member A (RhoA) inhibitor C3. As shown in Figure 7b, the induction of the three migration protein markers could be fully abrogated by AKT inhibitor LY294002, but not by ERK inhibitor PD98059 or RhoA inhibitor C3.

The involvement of FAK/AKT was also confirmed in HUVEC cells. As shown above in Fig. 4h, the angiogenesis markers KDR, eNOs, and EPHB4 were synergistically elevated following RXRα knockdown in combination with ERα overexpression. When the same transfected HUVEC cell was pretreated with AKT inhibitor LY294002, the elevation of the three differentiation markers was all efficiently abrogated (Fig. 7c).

Taken together, these results demonstrated that RXRα‘s inhibitory impacts on angiogenesis and migration were through affecting FAK and AKT pathways via phosphorylation.

## Discussion

Previous studies showed that ligand activations of RXR inhibited the carcinogenic process in leukemia, breast cancer, and other cancers^17–19^, but the underlying mechanism remains elusive. By comparing the impacts of RXRα in cancer cells with those in stem cells on differentiation, angiogenesis, and metabolism, we revealed that RXRα activated multiple cellular events contributing to tumor suppression.

RXRα level appears to be tightly controlled during differentiation. The fact that overexpression of RXRα in hMSCs did not promote differentiation but activated tumor suppressors (p53, p21, and p16) suggests that activation of RXRα during very early stages of differentiation may lead to harmful consequences, thus triggering the cellular defense mechanism. The senescence-like phenotypes caused by overexpression of RXRα is similar to previous studies with induction of excessive signaling of oncoproteins such as RAS^37^. Interestingly, the senescence-like response seems to occur only in the early stage of stem cell differentiation, overexpression in cancer cells (i.e., MCF-7) did not induce a significant increase of SA-β-galactosidase. The fact that RXRα can activate the senescence program revealed an important, yet not recognized, mechanism which may contribute to the anti-tumor effects of RXRα ligands found in clinical trials.

Compared to the elevated levels of RXRα in adult ECs or differentiated hMSCs, the RXRα levels in cancer cells were found to be much lower, similar to that of hMSCs before differentiation (Fig. 1). In connection with the cancer stem cell theory, does this suggest that the cellular mechanism to elevate RXRα be shut down at certain stages during cell differentiation in order for the transformation of stem cells into CSC to occur? Our data support this notion since suppressing RXRα could facilitate carcinogenesis-related features. For example, knockdown of RXRα in normal differentiated cells (HUVECs) could restore their capacity for migration and blood vessel formation (Fig. 5). In cancer cells, overexpressing RXRα not only inhibited cell proliferation, but also migration and angiogenesis (Fig. 3).

Due to its critical function, RXRα deficiency was shown to be embryonic lethal and RXRα expression is rarely lost in human tumors^19, 38^. However, the localization of RXRα had been shown to be altered in some cancers by sequestering it to the splicing factor compartments (SFCs), and this caused the loss of its activity^27^. Controlling the level of RXRα or suppressing its activity may be a key requirement during carcinogenesis. Whether this could help drive the transition from stem cell into CSC is an interesting question awaiting further study.

As a hallmark of cancer, the energy production in cancer cells is largely through glycolysis, similar to that of stem cells^1^. It is known that RXRα participates in lipid and glucose metabolism^12, 13, 16^. Here we demonstrated that RXRα overexpression in cancer cell lines interferes with their metabolism, suppressing enzymes involved in glycolysis which lead to lower rates of glucose utilization and lactate production as well as increased oxygen consumption (Fig. 6). These data revealed another important target of RXRα that is likely underlying its multiple tumor-suppressing functions. During differentiation, a key event occurring in stem cells is the reprogramming of the energy metabolism, shifting from glycolysis toward increased oxidative phosphorylation activity^39^. If cancer cells are originated from normal adult cells, it is difficult to understand why they would reprogram their glucose metabolism to glycolysis, which has ~18-fold lower efficiency for ATP production^1^. But if cancer cells are originated from stem cells, then the progression into cancer cells would not require alteration of the energy metabolism. In this regard, suppressing RXRα to prevent metabolism change is likely a prerequisite for carcinogenesis.

As to the cellular signaling underlying RXRα’s multiple impacts on differentiation, carcinogenesis, and metabolism, we found that RXRα‘s suppression on angiogenesis appears to be related to its inhibition of VEGF and the angiopoietin-like family member ANGPTL3. Our data also indicated that RXRα‘s anti-angiogenic function is at least partly through its interaction with ERα, and a synergistic effect was found that impacts differentiation markers. The downstream signal is transmitted via the PI3K-AKT pathway since all the effects of RXRα on cell differentiation, migration, and angiogenesis could be abrogated by inhibiting AKT. We demonstrated that RXRα overexpression caused phosphorylation of AKT and FAK. It has been shown that AKT stimulates histone acetylation that favors proliferation and tumor development, and phosphorylated AKT (S473) correlated with human gliomas and prostate tumors^40^. Phosphorylated FAK at Y925 is most likely responsible for the inhibition of RXRα on cell migration as FAK had been shown to stimulate migration^41^. The fact that RXRα-induced phosphorylation of AKT and FAK might as well be related to its inhibition of glycosylase since Akt/PI3K and RAS/FAK signaling correlates with an increase in glucose metabolism^42^.

As illustrated in Fig. 7d, our data revealed the multiple impacts of RXRα—on the one hand, inhibiting cell proliferation, angiogenesis, and migration, as well as promoting tumor-suppressing mechanism (senescence); on the other hand, regulating/participating in differentiation and metabolism reprogramming. These functions, which are at the crossroad between normal cell development and cancer transformation, make RXRα a possible key cellular component that affects the fate of stem cells between specialization/maturation and carcinogenesis.

Collectively, we presented here several lines of evidence showing that RXRα is fine-tuned during stem cell differentiation, and its ectopic expression could trigger multiple signaling pathways on tumor suppression. These results revealed an interesting cross talk between cell differentiation and carcinogenesis.

## Materials and methods

### Cell culture and treatment

Human bone marrow-derived MSCs (hBM-MSCs) were from Union Stem Cell and Gene Engineering Co. The purity of hMSCs was determined by flow cytometry analysis (positive for CD73 and CD105, negative for CD31 and KDR). To induce differentiation toward EC, hMSCs were cultured in differentiation medium (cc-4176, Lonza) supplemented with 50 ng/mL VEGF (PeproTech), 5 ng/mL basic fibroblast growth factor (bFGF) (PeproTech), and 2% fetal bovine serum (FBS) for 7 days. HUVECs were kindly provided by Tianjin Medical University. HMVEC, HAVECs, MCF-7, and Cos-7 were purchased from Shanghai Bio-Tech Co. HUVECs, HMVECs, HAVECs, MCF-7, and Cos-7 were cultured in DMEM/F12 medium supplemented with 10% fetal bovine serum at 37 °C in a humidified incubator with a 5% CO_2_ atmosphere.

### Plasmids, siRNA, and cell transfection

ERα and RXRα expression plasmids were constructed by subcloning into pcDNA3.1. The primers used for the amplification were listed in supporting information Table 2. For short-term depletion experiments, MSCs, MCF-7, and HUVECs were transfected with siRNA oligonucleotides (supporting information Table 3) using Lipofectamine 2000 (Invitrogen). Transfection reporter assays were performed in 6-well plates. MSCs, HUVECs, and MCF-7 cells were cultured in growth medium without antibiotics at 60% confluence for 2 days. MSCs were then transfected using the FuGENEHD transfection reagent (Roche); HUVECs and MCF-7 cells were transfected using the TurboFect transfection reagent (Thermo). After 48 h, cells were used for testing at mRNA and protein levels.

### MTT assay and EdU cell proliferation detection

Cell viability was examined by 3-(4,5-dimethylthiazol-2-yl)-2,5-diphenyltetrazolium (MTT) assay (Sigma). The absorbance of each well was measured using a Synergy™ 4 plate reader (Bioteck) with a test wavelength at 490 nm and a reference wavelength set at 630 nm. Absorbance is directly proportional to the number of survival cells.

EdU staining was conducted using Cell-Light EdU Apollo 488 in vitro kit (Ribobio), according to the manufacturer’s protocol. A total of 1 × 10^5^ cells were incubated with 50 μM EdU for 2 h. After fixation and permeabilization, the incorporated EdU was visualized by means of a click reaction using Alexa Fluor 488 azide (30 min, room temperature (RT)). The nuclear DNA was stained with DAPI (30 min, RT). The images were observed using confocal laser scanning microscope, and quantification was performed by counting the positively stained cells in five randomly selected areas under microscope.

### In vitro angiogenesis assay

Capillary tube formation of HUVECs and MCF-7 was induced using basement membrane-like material (EC Matrix TM; BD) as previously described^43^. Briefly, basement membrane-like material was diluted to 0.5–0.7 mg/mL in DMEM/F12 medium. A total of 5 × 10^4^ cells were seeded in 300 μL of 0.5–0.7 mg/mL Matrigel in each well of a 24-well plate. The Matrigel cell suspension was polymerized for 4 h at 37 °C. Then, 300 μL of DMEM/F12 medium supplemented with 50 ng/mL VEGF was added, and the gel-embedded cells were cultured at 37 °C and 4% CO_2_. The structures were photographed using a phase contrast microscope (Olympus) after 48 h. Total cord length was quantified using image-Pro Plus v4.5 software.

### Cell migration assay

MSCs, HUVECs, and MCF-7 grown in 6-well plates were transfected with RXRα and then wounded using a sterile pipette tip as described in ref. ^25^. The progress of migration was photographed immediately following injury, and micrographs were taken at 0, 24, and 48 h.

### Transwell chamber assay

After MCF-7 cells were transfected with RXRα, cells were harvested by trypsin, and 1.0 × 10^4^ cells in 200 μL of 1% FBS-containing medium were then seeded into the upper chamber of a transwell cell culture insert as previously described^26^. The lower chamber was filled with 600 μL of medium containing 10% FBS. Twenty-four hours later, the cells in the upper chamber were removed using a cotton swab. Cells that had migrated to the lower side of the membrane were fixed with 4% paraformaldehyde and stained with DAPI. The number of migrated cells was counted and photographed in five fields (the upper, lower, left, right, and middle) under microscope, and the average number was obtained from three independent experiments.

### Detection of senescence-associated β-galactosidase

SA-β-gal was detected according to Dimri et al.^22, 23^. After hMSCs transfected with RXRα for 48 h, cells were fixed with 4% formaldehyde, washed and exposed overnight at 37 °C to a solution containing 1 mg/mL 5-bromo-4-chloro-3-indolyl-β-galactopyranoside, 5 mM potassium ferrocyanide, 5 mM potassium ferricyanide, 150 mM NaCl, 2 mM MgCl_2_, and 0.1 M phosphate buffer, pH 6.0. Then, the staining was photographed using a phase contrast microscope (Olympus).

### Quantitative real-time RT-PCR (qRT-PCR)

Total RNA was isolated from cells using Trizol reagent (Invitrogen), reverse-transcribed complementary DNA was synthesized with random primers or microRNAs specific stem-loop primers. qRT-PCR analysis was performed using Fast SYBR Green Master Mix (Applied Biosystems) in a Biosystems StepOneTM Real-Time PCR machine (Applied Biosystem, CA). Glyceraldehyde-3-phosphate dehydrogenase (GAPDH) was used as internal controls. The primers used for qRT-PCR were listed in supporting information Table 1.

### Western blotting

After lysing cells with extraction buffer, cell extracts were separated by SDS-PAGE and then transferred onto nitrocellulose membranes. The following primary antibodies were used: rabbit anti-eNOS, KDR, Cyr61, Myl9, MMP9, ANGPTL3, and VEGF (abcam); EPHB4, RXRα, ERα, pAKT, AKT, pY925 FAK, FAK, pERK, ERK, and mouse anti-GAPDH (Santa Cruz Biotechnology). Antibody incubations were performed overnight at 4 °C. The secondary antibodies used were IRDyeTM-800-conjugated anti-mouse and anti-rabbit IgG (Li-COR Biosciences). Immunoreactivity was detected using an Odyssey Infrared Imaging System (Gene Company Limited). All immunoblots were repeated at least two–three times.

### Immunoprecipitation (IP) analysis

HUVEC cells were co-transfected with RXRα and ERα. Protein extracts were isolated from cells using RIPA buffer. Tagged proteins were immunoprecipitated overnight at 4 °C using protein A/G agarose (CW0349) and anti-RXRα antibody, and then all complexes were pelleted at 3000 rpm for 3 min. The beads were washed and fractionated by 12% SDS-PAGE, followed by transfer to a nitrocellulose membrane. The membrane was immunoblotted with mouse anti-ERα (1:5000) and β-actin (1:250) overnight at 4 °C and then incubated with anti-mouse secondary antibodies (Li-COR Biosciences) for 1 h at RT. The specific proteins were visualized by Odyssey Infrared Imaging System (Gene Company Limited).

### Immunocytochemistry

The cells were fixed in 4% paraformaldehyde for 15 min, then blocked with normal goat serum for 20 min at room temperature. After incubation with rabbit anti-RXRα (santa cruz) and ERa (santa cruz) in a humid chamber overnight, cells were incubated with appropriate secondary antibodies (fluorescein isothiocyanate (FITC)-labeled goat anti-rabbit IgG) for 30 min at 37 °C. Washing with PBS, and then the samples were observed under laser scanning confocal microscope (OLYMPUS). DAPI stain (blue) high lights the total nuclei.

### Luciferase assay

Luciferase activity assay was performed using the Luciferase Assay System (Promega) according to the instructions. Briefly, HUVEC cells were seeded in 24-well plates, ERa reporter plasmids (ERa-2235-Luc) were co-transfected with sh-RXRα plasmids or control (pSUPER) plasmids using Lipofectamine 2000 for 24 h. ERa promoter plasmids and RXRα expression plasmids and/or ERa expression plasmids were co-transfected into COS-7 cells using Lipofectamine 2000 for 24 h. The transfected cells were lysed in Cell Culture Lysis Reagent. An aliquot of 20 μL of cell lysate was added into a 96-well enzyme label plate and reading was initiated by the injection of 100 μL of Luciferase Assay Reagent into the plate on a Synergy™4 (Bioteck). Transfection efficiencies were normalized by total protein concentration of each luciferase assay preparation.

### Statistical analysis

Data were expressed as the mean ± SE, accompanied by the number of experiments performed independently, and analyzed by *t*-test. Differences with *P* < 0.05 were considered statistically significant.

## Electronic supplementary material

SI Table and Legend

Supplemental Figure S1
