# Supplementary material for: RXRα provokes tumor suppression through p53/p21/p16 and PI3K-AKT signaling pathways during stem cell differentiation and in cancer cells
Source: Cell Death Dis. 2018 May 10;9(5):532. doi: 10.1038/s41419-018-0610-1 (PMC5945609; doi:10.1038/s41419-018-0610-1)
Supplement: Supplementary file 1 — SI Table and Legend [file 41419_2018_610_MOESM1_ESM.doc]

Table1: Primers for qPCR

| Gene | Forward | Reverse |
| --- | --- | --- |
| P16 | CTTCCTGGACACGCTGGT | GGGATGTCTGAGGGACCTT |
| P53 | TCAACAAGATGTTTTGCCAACTG | ATGTGCTGTGACTGCTTGTAGATG |
| P21 | CAGGGGACAGCAGAGGAAGA | TTAGGGCTTCCTCTTGGAGAA |
| Cyr61 | TTAGTCGTCACCCTTCTC | TCCCCGTTTTGGTAGATTC |
| Myl9 | GAAGAAACAGGCCAGGAGA | GTGTAGGGAAGGGGACTGAGAGG |
| MMP9 | CAAGGATGGTCTACTGGCACA | CGAAGATGAATGGAAATACGC |
| EphB4 | GATGCCTGGAGTTACGGGATTG | TCCAGCATGAGCTGGTGGAG |
| KDR | TTACTATTCCCAGCTACATGATCAG | AGACGGACTCAGAACCACATCATAA |
| eNOS | GGCATCACCAGGAAGAACACC | TCACTCGCTTCGCCATCAC |
| ERa | CCTACTACCTGGAGAACGAG | CTCTTCGGTCTTTTCGTATG |
| HK2 | CTGGACCCGACTCAGGAGGACT | CCTCGCCTTTGTTCTCCTTGAT |
| ALDOA | CGGGAAGAAGGAGAACCTG | GACCGCTCGGAGTGTACTTT |
| TPI1 | ACAGCCATAGTTGATCCAGCT | CCTCAACAGACTCTTCACTAGT |
| PDK1 | TCTGGCTGGTTTTGGTTATGGATTG | CCTCGTGGTTGGTGTTGTAATGC |
| PGAM1 | CCTCCTGTGAGAGCCTGAAG | CTTCTTCACCTTGCCCTGAG |
| ENO1 | CGCGGATCCATGTCTATTCTCAAG | TGTCGACTGCCCACAGCTTACTTG |
| PKM2 | GTCATTCATCCGCAAGGCATCTG | GCACCGTCCAATCATCATCTTCTG |
| LDHA | GATTCAGCCCGATTCCGTTACC | AGAGACACCAGCAACATTCATTCC |
| RXRa | TCCTTCTCCCACCGCTCCATC | CAGCTCCGTCTTGTCCATCTG |
| GAPDH | TCAACGGCACAGTCAAGG | AGAAGGGGCGGAGATGA |

**Table2: Primers used for construction of** plasmids

| plasmid | Forward | Reverse |
| --- | --- | --- |
| RXRα | GGACACGCGTATGCGGATGGATAAGT | TACACTCGAGGAAGAGGAGTTTCAGG |
| ERα | GACAACGCGTATGAGGACTGCCTGAT | GAGGCTCGAGGAACCGTCTTTCACAT |

**Table3: siRNA sequences were used**

| siRXRα | AACUGAUGUACAGUAGACG |
| --- | --- |
| Control siRNA | AUCUTCGCATAUGUCGTGU |

**Figure S-1 Legend:**

RXRα expression level in various cancer cell lines. The expression was determined by qRT-PCR and Western blotting (*P*<0.01, n=3), hMSC control are cells cultured in differentiation medium (EDM) at day 7, all cancer cell lines were cultured in DMEM/F12 medium supplemented with 10% FBS, MCF10a cell line were cultured in F12 medium supplemented with 5% Horse Serum.
