# Supplementary figures and images for: RXRα provokes tumor suppression through p53/p21/p16 and PI3K-AKT signaling pathways during stem cell differentiation and in cancer cells
Source: Cell Death Dis. 2018 May 10;9(5):532. doi: 10.1038/s41419-018-0610-1 (PMC5945609; doi:10.1038/s41419-018-0610-1)

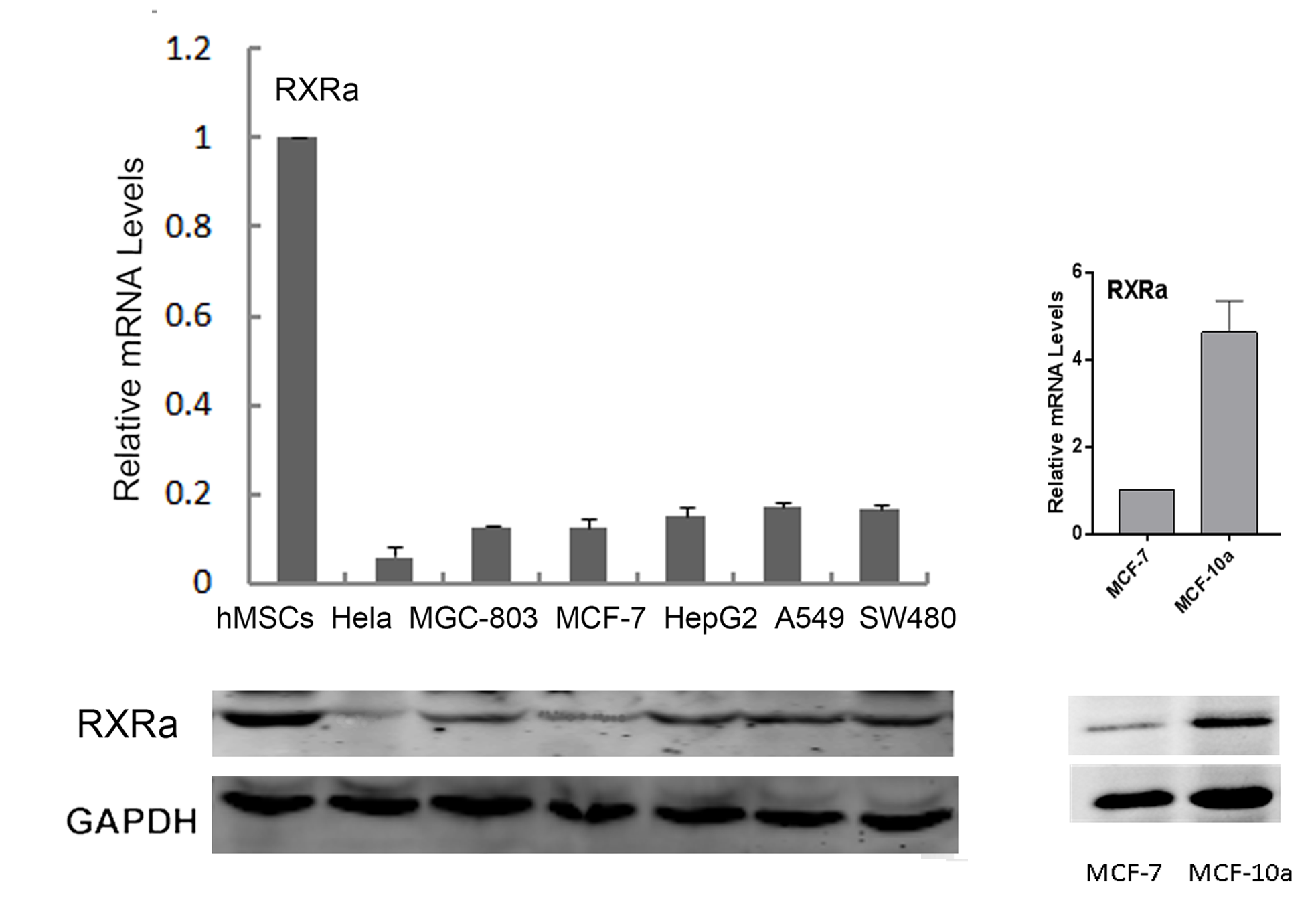

Supplement: Supplementary file 2 — Supplemental Figure S1 [file 41419_2018_610_MOESM2_ESM.tif]
